# Supplementary material for: A Likelihood Approach for Real-Time Calibration of Stochastic Compartmental Epidemic Models
Source: PLoS Comput Biol. 2017 Jan 17;13(1):e1005257. doi: 10.1371/journal.pcbi.1005257 (PMC5240920; doi:10.1371/journal.pcbi.1005257)
Supplement: S1 File — (TAR.GZ) [file pcbi.1005257.s014.tar.gz › HSPH_Online-SI-Revision/MSS10/n10-mild/n10-mild_NEW2_table8.pdf]

1	newly 3 weeks spec	60.	55.66	{10., 107.}	52.1	0.345917	newly 3 weeks cumul	239.	224.47	{64., 351.}	42.0126	0.236291	newly 5 weeks spec	13.	28.58	{0., 61.}	58.9492	0.716839	newly 5 weeks cumul	350.	292.98	{82., 478.}	40.6171	0.253476	newly 42 weeks cumul	920.	366.08	{88., 644.}	60.6022	0.504942
2	newly 3 weeks spec	35.	24.46	{9., 43.}	40.9714	0.260785	newly 3 weeks cumul	125.	117.03	{66., 169.}	26.984	0.13394	newly 5 weeks spec	9.	9.25	{0., 20.}	59.	0.561887	newly 5 weeks cumul	172.	141.11	{71., 211.}	28.5291	0.15898	newly 47 weeks cumul	189.	158.29	{71., 231.}	29.1481	0.163014
3	newly 3 weeks spec	49.	25.2	{5., 50.}	52.5714	0.457395	newly 3 weeks cumul	214.	138.38	{63., 216.}	38.1869	0.25137	newly 5 weeks spec	21.	9.18	{0., 25.}	67.1429	0.89411	newly 5 weeks cumul	265.	162.27	{65., 273.}	41.9057	0.286485	newly 50 weeks cumul	281.	176.3	{65., 289.}	42.3559	0.292284
4	newly 3 weeks spec	42.	24.68	{11., 40.}	44.8571	0.301221	newly 3 weeks cumul	187.	114.81	{76., 155.}	39.	0.232549	newly 5 weeks spec	18.	11.42	{2., 22.}	51.1111	0.429767	newly 5 weeks cumul	235.	142.14	{88., 204.}	39.9915	0.242303	newly 45 weeks cumul	289.	164.09	{96., 247.}	43.9343	0.277885
5	newly 3 weeks spec	82.	54.59	{30., 76.}	36.1829	0.216548	newly 3 weeks cumul	270.	192.07	{130., 255.}	29.8259	0.16458	newly 5 weeks spec	96.	41.18	{18., 63.}	57.3333	0.434453	newly 5 weeks cumul	451.	283.33	{185., 382.}	37.3681	0.217986	newly 25 weeks cumul	1188.	506.94	{286., 778.}	57.3283	0.404544
6	newly 3 weeks spec	58.	42.07	{10., 82.}	48.6034	0.386932	newly 3 weeks cumul	223.	198.07	{83., 329.}	36.991	0.215168	newly 5 weeks spec	18.	15.61	{0., 40.}	75.2778	0.697179	newly 5 weeks cumul	288.	237.25	{102., 401.}	38.5799	0.23807	newly 51 weeks cumul	298.	263.15	{106., 465.}	41.5872	0.240179
7	newly 3 weeks spec	72.	22.	{2., 41.}	69.8333	0.731851	newly 3 weeks cumul	231.	106.8	{34., 173.}	55.368	0.427429	newly 5 weeks spec	20.	10.88	{0., 28.}	71.1	0.900105	newly 5 weeks cumul	292.	133.62	{34., 224.}	55.8151	0.443418	newly 47 weeks cumul	336.	151.01	{38., 295.}	56.3006	0.463061
8	newly 3 weeks spec	137.	44.13	{14., 77.}	67.7883	0.597339	newly 3 weeks cumul	446.	208.6	{88., 323.}	53.2287	0.383122	newly 5 weeks spec	94.	16.77	{1., 34.}	82.1596	1.02417	newly 5 weeks cumul	661.	253.43	{113., 376.}	61.6596	0.47245	newly 42 weeks cumul	1278.	297.54	{133., 464.}	76.7183	0.697758
9	newly 3 weeks spec	36.	23.96	{9., 44.}	44.4444	0.335877	newly 3 weeks cumul	178.	113.72	{54., 180.}	38.8989	0.248057	newly 5 weeks spec	6.	10.08	{0., 24.}	125.	0.599445	newly 5 weeks cumul	197.	139.46	{63., 234.}	37.4213	0.231991	newly 46 weeks cumul	197.	158.99	{68., 266.}	39.3452	0.225617
10	newly 3 weeks spec	48.	23.31	{1., 43.}	58.6458	0.629836	newly 3 weeks cumul	183.	111.7	{41., 210.}	47.9563	0.33247	newly 5 weeks spec	54.	11.86	{0., 30.}	79.037	1.32904	newly 5 weeks cumul	294.	139.8	{41., 268.}	55.4966	0.436485	newly 43 weeks cumul	610.	159.93	{41., 289.}	73.782	0.700071
11	newly 3 weeks spec	38.	37.4	{0., 85.}	79.5263	0.705452	newly 3 weeks cumul	143.	210.22	{26., 398.}	91.8462	0.380483	newly 5 weeks spec	2.	14.68	{0., 46.}	734.	1.1199	newly 5 weeks cumul	158.	247.66	{26., 522.}	100.468	0.400966	newly 48 weeks cumul	209.	264.31	{26., 549.}	80.6172	0.403336
12	newly 3 weeks spec	4.	6.68	{0., 20.}	177.5	1.03004	newly 3 weeks cumul	21.	41.69	{8., 96.}	129.19	0.35857	newly 5 weeks spec	0.1	1.77	{0., 6.}	1828.	0.367785	newly 5 weeks cumul	21.	46.76	{8., 111.}	152.857	0.389401	newly 50 weeks cumul	21.	48.68	{8., 114.}	162.	0.397539
13	newly 3 weeks spec	14.	22.96	{8., 38.}	83.5714	0.281113	newly 3 weeks cumul	103.	112.05	{66., 163.}	28.3592	0.12252	newly 5 weeks spec	15.	10.8	{1., 21.}	46.4	0.431325	newly 5 weeks cumul	131.	138.49	{72., 204.}	28.2214	0.125807	newly 42 weeks cumul	149.	157.26	{72., 237.}	31.8255	0.141591
14	newly 3 weeks spec	78.	55.5	{35., 77.}	31.0769	0.178853	newly 3 weeks cumul	298.	228.7	{166., 287.}	23.9463	0.126806	newly 5 weeks spec	48.	31.54	{15., 50.}	38.0833	0.242968	newly 5 weeks cumul	412.	302.69	{212., 389.}	26.9684	0.146688	newly 43 weeks cumul	587.	389.13	{264., 550.}	34.3595	0.198863
15	newly 3 weeks spec	142.	83.69	{38., 126.}	41.5563	0.279153	newly 3 weeks cumul	509.	372.46	{224., 506.}	28.6248	0.168562	newly 5 weeks spec	97.	35.8	{9., 67.}	63.732	0.601446	newly 5 weeks cumul	755.	462.99	{273., 636.}	38.947	0.241471	newly 45 weeks cumul	1453.	530.98	{315., 764.}	63.4563	0.471913
16	newly 3 weeks spec	54.	45.45	{18., 73.}	36.4259	0.206656	newly 3 weeks cumul	234.	217.07	{141., 310.}	23.6197	0.114052	newly 5 weeks spec	35.	20.08	{5., 38.}	46.6857	0.420623	newly 5 weeks cumul	327.	266.9	{165., 393.}	25.9266	0.139589	newly 46 weeks cumul	351.	299.89	{180., 431.}	25.5128	0.135021
17	newly 3 weeks spec	90.	65.25	{0., 139.}	52.3889	0.575332	newly 3 weeks cumul	301.	237.68	{30., 431.}	44.7309	0.353019	newly 5 weeks spec	86.	42.54	{0., 100.}	62.0698	0.885826	newly 5 weeks cumul	488.	334.43	{30., 646.}	46.6373	0.4127	newly 43 weeks cumul	2262.	464.79	{30., 962.}	79.4523	0.924372
18	newly 3 weeks spec	41.	44.92	{15., 80.}	50.0488	0.261651	newly 3 weeks cumul	174.	187.69	{90., 284.}	35.0517	0.1679	newly 5 weeks spec	45.	25.14	{3., 48.}	55.2	0.549783	newly 5 weeks cumul	264.	247.37	{100., 377.}	33.9962	0.187102	newly 43 weeks cumul	314.	324.91	{133., 553.}	42.0541	0.211032
19	newly 3 weeks spec	42.	14.92	{0., 39.}	64.8095	0.89303	newly 3 weeks cumul	145.	80.57	{15., 143.}	47.3034	0.359993	newly 5 weeks spec	27.	3.4	{0., 11.}	87.4074	1.62009	newly 5 weeks cumul	208.	91.75	{15., 170.}	56.4567	0.499494	newly 46 weeks cumul	266.	96.26	{15., 178.}	63.8195	0.588099
20	newly 3 weeks spec	94.	26.02	{1., 57.}	73.3404	0.921616	newly 3 weeks cumul	348.	190.64	{51., 310.}	46.431	0.345425	newly 5 weeks spec	48.	5.89	{0., 18.}	88.3125	1.86767	newly 5 weeks cumul	453.	209.44	{61., 353.}	53.9205	0.420724	newly 47 weeks cumul	551.	213.52	{61., 360.}	61.2486	0.499402
21	newly 3 weeks spec	33.	20.12	{4., 38.}	48.8485	0.399355	newly 3 weeks cumul	145.	95.63	{45., 143.}	38.9448	0.249038	newly 5 weeks spec	16.	8.3	{0., 18.}	62.625	0.818444	newly 5 weeks cumul	184.	116.05	{45., 193.}	41.3533	0.275804	newly 47 weeks cumul	205.	132.97	{45., 216.}	42.2585	0.286767
22	newly 3 weeks spec	79.	31.6	{3., 59.}	61.5443	0.633202	newly 3 weeks cumul	247.	145.37	{48., 242.}	45.0729	0.331182	newly 5 weeks spec	60.	13.48	{0., 31.}	77.5333	1.09136	newly 5 weeks cumul	392.	180.5	{54., 309.}	54.8622	0.435701	newly 43 weeks cumul	671.	204.63	{54., 355.}	69.5037	0.622794
23	newly 3 weeks spec	1.	9.7	{0., 27.}	940.	1.00565	newly 3 weeks cumul	20.	57.46	{10., 112.}	208.2	0.461549	newly 5 weeks spec	0.1	3.48	{0., 11.}	3522.	0.553154	newly 5 weeks cumul	20.	66.85	{10., 139.}	255.15	0.503834	newly 48 weeks cumul	20.	70.6	{10., 152.}	273.9	0.515312
24	newly 3 weeks spec	31.	34.69	{0., 84.}	92.871	0.818946	newly 3 weeks cumul	126.	144.37	{21., 311.}	71.4206	0.379059	newly 5 weeks spec	21.	15.53	{0., 44.}	89.381	1.19298	newly 5 weeks cumul	167.	182.69	{21., 371.}	70.485	0.407984	newly 44 weeks cumul	190.	209.99	{21., 444.}	73.5737	0.428354
25	newly 3 weeks spec	26.	36.74	{1., 71.}	92.6923	0.533532	newly 3 weeks cumul	124.	183.67	{45., 324.}	77.9435	0.283346	newly 5 weeks spec	3.	18.14	{0., 45.}	576.	1.04259	newly 5 weeks cumul	138.	228.35	{45., 391.}	94.0652	0.314709	newly 51 weeks cumul	140.	261.91	{45., 500.}	115.564	0.349438
26	newly 3 weeks spec	39.	15.48	{0., 38.}	68.2051	0.871288	newly 3 weeks cumul	98.	91.1	{28., 165.}	46.5102	0.25069	newly 5 weeks spec	10.	5.32	{0., 18.}	87.4	1.23125	newly 5 weeks cumul	122.	105.79	{31., 200.}	47.9262	0.276984	newly 42 weeks cumul	155.	112.93	{31., 223.}	51.129	0.332255
27	newly 3 weeks spec	6.	15.96	{2., 35.}	193.667	0.481688	newly 3 weeks cumul	37.	79.24	{29., 131.}	122.324	0.315528	newly 5 weeks spec	1.	7.74	{0., 17.}	718.	0.88129	newly 5 weeks cumul	40.	98.1	{34., 172.}	152.25	0.359417	newly 44 weeks cumul	46.	113.07	{34., 205.}	155.848	0.362579
28	newly 3 weeks spec	58.	42.02	{6., 80.}	45.3103	0.378089	newly 3 weeks cumul	196.	158.37	{44., 251.}	36.852	0.238782	newly 5 weeks spec	40.	23.6	{0., 55.}	56.75	0.679517	newly 5 weeks cumul	279.	212.74	{44., 363.}	38.1075	0.264084	newly 22 weeks cumul	839.	289.32	{44., 502.}	65.5518	0.57754
29	newly 3 weeks spec	51.	26.7	{8., 44.}	49.098	0.43696	newly 3 weeks cumul	209.	136.	{66., 196.}	36.6316	0.241609	newly 5 weeks spec	29.	10.54	{0., 22.}	67.4483	0.81731	newly 5 weeks cumul	281.	163.53	{78., 244.}	42.5374	0.294232	newly 45 weeks cumul	388.	186.28	{78., 304.}	52.8351	0.3897
30	newly 3 weeks spec	44.	15.31	{3., 29.}	65.5227	0.64823	newly 3 weeks cumul	129.	82.85	{27., 141.}	41.2481	0.282553	newly 5 weeks spec	32.	5.69	{0., 15.}	82.9688	1.28247	newly 5 weeks cumul	194.	98.09	{32., 168.}	51.2216	0.380615	newly 44 weeks cumul	268.	105.9	{34., 178.}	60.7761	0.488496
31	newly 3 weeks spec	39.	41.12	{4., 76.}	58.9231	0.414433	newly 3 weeks cumul	120.	178.67	{48., 294.}	74.4583	0.272936	newly 5 weeks spec	8.	21.35	{0., 46.}	218.375	0.75148	newly 5 weeks cumul	150.	226.84	{60., 366.}	78.5467	0.290266	newly 51 weeks cumul	165.	268.68	{61., 479.}	91.7455	0.319221
32	newly 3 weeks spec	110.	51.88	{16., 89.}	53.3636	0.412193	newly 3 weeks cumul	385.	223.01	{111., 330.}	42.7506	0.275608	newly 5 weeks spec	87.	24.72	{0., 50.}	71.6552	0.872634	newly 5 weeks cumul	596.	283.3	{141., 446.}	52.4664	0.365465	newly 24 weeks cumul	1460.	322.17	{156., 514.}	77.9336	0.707161
33	newly 3 weeks spec	26.	25.07	{13., 39.}	33.6538	0.169753	newly 3 weeks cumul	122.	119.78	{72., 169.}	22.623	0.103865	newly 5 weeks spec	8.	13.26	{2., 25.}	95.25	0.372642	newly 5 weeks cumul	152.	152.24	{87., 214.}	24.7632	0.113093	newly 45 weeks cumul	158.	174.37	{98., 263.}	32.2215	0.132917
34	newly 3 weeks spec	95.	23.45	{0., 51.}	75.3158	0.939324	newly 3 weeks cumul	318.	159.52	{38., 283.}	50.761	0.406182	newly 5 weeks spec	73.	5.12	{0., 14.}	92.9863	1.93905	newly 5 weeks cumul	481.	177.35	{38., 318.}	63.1289	0.545871	newly 45 weeks cumul	672.	180.98	{38., 324.}	73.0685	0.684718
35	newly 3 weeks spec	44.	32.66	{15., 58.}	38.1364	0.226159	newly 3 weeks cumul	154.	139.98	{86., 197.}	23.013	0.115606	newly 5 weeks spec	28.	15.85	{3., 31.}	52.1071	0.428477	newly 5 weeks cumul	217.	178.67	{106., 248.}	26.7235	0.143901	newly 45 weeks cumul	280.	217.22	{125., 314.}	31.15	0.174529
36	newly 3 weeks spec	9.	12.45	{0., 29.}	111.889	0.676897	newly 3 weeks cumul	71.	72.19	{15., 140.}	55.6761	0.293268	newly 5 weeks spec	1.	3.86	{0., 13.}	390.	0.858121	newly 5 weeks cumul	72.	83.23	{15., 159.}	66.9028	0.315155	newly 47 weeks cumul	75.	88.01	{15., 169.}	70.7333	0.32804
37	newly 3 weeks spec	26.	14.53	{0., 38.}	77.8077	1.19573	newly 3 weeks cumul	60.	82.23	{10., 188.}	87.75	0.37773	newly 5 weeks spec	12.	4.47	{0., 15.}	99.75	1.60441	newly 5 weeks cumul	86.	94.2	{10., 210.}	74.6279	0.40803	newly 50 weeks cumul	182.	101.02	{10., 219.}	61.6593	0.551652
38	newly 3 weeks spec	105.	42.79	{15., 71.}	59.2667	0.455018	newly 3 weeks cumul	339.	192.7	{106., 266.}	43.1563	0.273321	newly 5 weeks spec	72.	17.58	{2., 36.}	75.7222	0.853253	newly 5 weeks cumul	490.	238.98	{130., 349.}	51.2286	0.34513	newly 46 weeks cumul	831.	275.22	{142., 402.}	66.8809	0.518003
39	newly 3 weeks spec	5.	7.23	{0., 19.}	135.	0.860856	newly 3 weeks cumul	20.	40.21	{7., 80.}	132.75	0.388603	newly 5 weeks spec	0.1	1.88	{0., 6.}	1930.	0.430074	newly 5 weeks cumul	20.	45.6	{7., 93.}	159.7	0.421226	newly 47 weeks cumul	20.	46.6	{7., 93.}	164.7	0.425606
40	newly 3 weeks spec	69.	37.12	{15., 57.}	46.7826	0.321701	newly 3 weeks cumul	225.	135.59	{84., 180.}	40.12	0.241593	newly 5 weeks spec	51.	27.86	{6., 53.}	50.4706	0.424188	newly 5 weeks cumul	346.	194.95	{102., 272.}	44.0549	0.280074	newly 20 weeks cumul	809.	329.9	{131., 545.}	59.4091	0.448924
41	newly 3 weeks spec	0.1	3.42	{0., 15.}	3470.	0.478584	newly 3 weeks cumul	0.1	32.8	{3., 85.}	32700.	2.28548	newly 5 weeks spec	0.1	0.76	{0., 0.}	850.	0.106536	newly 5 weeks cumul	0.1	35.55	{3., 95.}	35450.	2.29943	newly 51 weeks cumul	0.1	35.97	{3., 95.}	35870.	2.30143
42	newly 3 weeks spec	35.	34.44	{9., 64.}	47.8857	0.289267	newly 3 weeks cumul	180.	149.79	{73., 229.}	32.5833	0.189136	newly 5 weeks spec	44.	17.87	{0., 37.}	61.8409	0.728092	newly 5 weeks cumul	253.	193.07	{85., 315.}	35.8696	0.222982	newly 43 weeks cumul	319.	234.52	{94., 395.}	37.4922	0.243141
43	newly 3 weeks spec	0.1	4.05	{0., 12.}	4094.	0.533814	newly 3 weeks cumul	1.	33.69	{3., 86.}	3269.	1.23881	newly 5 weeks spec	0.1	1.02	{0., 0.}	1104.	0.144226	newly 5 weeks cumul	1.	35.97	{3., 96.}	3497.	1.2505	newly 49 weeks cumul	1.	37.17	{3., 99.}	3617.	1.25559
44	newly 3 weeks spec	90.	51.17	{22., 83.}	47.1222	0.315366	newly 3 weeks cumul	310.	235.2	{147., 341.}	29.5484	0.164416	newly 5 weeks spec	69.	20.95	{2., 41.}	69.7536	0.726457	newly 5 weeks cumul	468.	288.82	{164., 415.}	39.7051	0.24322	newly 45 weeks cumul	766.	331.7	{183., 480.}	57.2115	0.399624
45	newly 3 weeks spec	7.	5.4	{0., 16.}	95.4286	1.04204	newly 3 weeks cumul	18.	38.1	{6., 82.}	141.889	0.386801	newly 5 weeks spec	3.	1.48	{0., 6.}	118.	1.28594	newly 5 weeks cumul	26.	42.19	{6., 98.}	106.346	0.370423	newly 52 weeks cumul	26.	43.12	{6., 101.}	109.923	0.374399
46	newly 3 weeks spec	14.	16.23	{0., 40.}	86.3571	0.598566	newly 3 weeks cumul	72.	86.57	{21., 165.}	65.3194	0.29615	newly 5 weeks spec	4.	5.93	{0., 15.}	147.25	0.95961	newly 5 weeks cumul	77.	101.14	{21., 202.}	74.6494	0.310591	newly 49 weeks cumul	79.	111.69	{21., 220.}	83.8608	0.327166
47	newly 3 weeks spec	62.	37.12	{16., 65.}	45.0323	0.308986	newly 3 weeks cumul	217.	181.2	{107., 251.}	25.659	0.13572	newly 5 weeks spec	38.	13.6	{2., 29.}	65.3158	0.697372	newly 5 weeks cumul	302.	218.74	{123., 325.}	31.5762	0.183615	newly 49 weeks cumul	355.	242.81	{127., 369.}	35.5634	0.213658
48	newly 3 weeks spec	10.	23.62	{0., 50.}	182.8	0.662738	newly 3 weeks cumul	99.	131.86	{35., 234.}	69.0303	0.276467	newly 5 weeks spec	18.	5.6	{0., 19.}	81.3333	1.38699	newly 5 weeks cumul	143.	148.77	{36., 266.}	54.5804	0.280411	newly 46 weeks cumul	178.	155.01	{36., 277.}	47.9944	0.293594
49	newly 3 weeks spec	135.	52.69	{16., 88.}	61.5481	0.513943	newly 3 weeks cumul	399.	229.49	{110., 345.}	44.6642	0.297081	newly 5 weeks spec	100.	24.58	{1., 57.}	75.42	0.922287	newly 5 weeks cumul	612.	290.89	{128., 431.}	52.9003	0.377743	newly 42 weeks cumul	1281.	345.38	{141., 550.}	73.0383	0.634975
50	newly 3 weeks spec	34.	24.99	{3., 55.}	52.5588	0.470993	newly 3 weeks cumul	108.	118.2	{41., 199.}	48.0185	0.228506	newly 5 weeks spec	11.	11.74	{0., 31.}	97.6364	0.848963	newly 5 weeks cumul	144.	147.27	{45., 268.}	45.7986	0.235877	newly 42 weeks cumul	204.	169.99	{45., 303.}	45.9461	0.28256
